# An Assessment of Computer-Generated Stimuli for Use in Studies of Body Size Estimation and Bias

**DOI:** 10.3389/fpsyg.2019.02390

**Published:** 2019-10-22

**Authors:** Joanna Alexi, Kendra Dommisse, Dominique Cleary, Romina Palermo, Nadine Kloth, Jason Bell

**Affiliations:** ^1^School of Psychological Science, University of Western Australia, Perth, WA, Australia; ^2^Telethon Kids Institute, University of Western Australia, Perth, WA, Australia

**Keywords:** computer-generated bodies, body size estimation, biases, regression to the mean, serial dependence, body image disturbance

## Abstract

Inaccurate body size judgments are associated with body image disturbances, a clinical feature of many eating disorders. Accordingly, body-related stimuli have become increasingly important in the study of estimation inaccuracies and body image disturbances. Technological advancements in the last decade have led to an increased use of computer-generated (CG) body stimuli in body image research. However, recent face perception research has suggested that CG face stimuli are not recognized as readily and may not fully tap facial processing mechanisms. The current study assessed the effectiveness of using CG stimuli in an established body size estimation task (the “bodyline” task). Specifically, we examined whether employing CG body stimuli alters body size judgments and associated estimation biases. One hundred and six 17- to 25-year-old females completed the CG bodyline task, which involved estimating the size of full-length CG body stimuli along a visual analogue scale. Our results show that perception of body size for CG stimuli was non-linear. Participants struggled to discriminate between extreme bodies sizes and overestimated the size change between near to average bodies. Furthermore, one of our measured size estimation biases was larger for CG stimuli. Our collective findings suggest using caution when employing CG stimuli in experimental research on body perception.

## Introduction

Eating disorders are an increasingly prevalent health concern. It is estimated that 15% of Australian women have experienced an eating disorder during their lifetime, which has necessitated clinical intervention (The Butterfly Foundation, 2012). Eating disorders are generally considered as encompassing a pervasive disturbance of eating and/or eating-related behaviors that lead to a disordered consumption or absorption of food and substantial impairments in mental or physical health (American Psychiatric Association, 2013). One of the core diagnostic features in eating disorders, such as anorexia nervosa, is a disturbance in the way one’s body is experienced (American Psychiatric Association, 2013). Additionally, body image disturbance is a clinical marker of many other subtypes of eating disorders too, such as bulimia nervosa and binge-eating disorder (Zanetti et al., 2013; Lewer et al., 2016), and it can be very difficult to treat, even following recovery (Eshkevari et al., 2014). It is not surprising then, that a substantial amount of research has been dedicated to understanding the mechanisms underlying body image disturbance (for a review of some of the identified mechanisms, see Cash and Deagle, 1997; Gaudio et al., 2014; Riva and Dakanalis, 2018). Regarding the perceptual component of body image disturbance, it has been found that individuals with anorexia nervosa and bulimia nervosa tend to overestimate their body size more than healthy controls (Vocks et al., 2007; Gardner and Brown, 2014). Several research groups have sought to identify potentially underlying perceptual biases and distortions, such as adaptation, regression to the mean, and serial dependence biases, which may be linked to these body image disturbances (see: Cornelissen et al., 2016; Mohr et al., 2016; Sturman et al., 2017; Alexi et al., 2018, 2019). Within this literature, there have been considerable variations in the types of body stimuli used to study judgments of body size and weight.

Early on, methodologies involved the use of schematic drawings of participants’ estimated size (Slade, 1985), distorting mirror techniques (Traub and Orbach, 1964), and silhouette methods (Bell et al., 1986; Skrzypek et al., 2001). Researchers then began to use image and video distortion techniques that involved adjusting body image widths to produce a body size change (Slade, 1985; Taylor and Cooper, 1992; Skrzypek et al., 2001). However, it has now been established that horizontal stretching not only gives the body an unrealistic appearance, but can also preserve key size markers in the original image, such as hip-to-waist ratios, which may impact the validity of findings (Dondzilo et al., 2018).

More recently, the advancement and accessibility of photographic and computer graphic technology have led to an increased use of real and computer-generated (CG) body images, which vary along the continuum, in the study of body image distortion. Real body stimuli have been used in a broad range of body image-related studies (e.g., Blechert et al., 2010; Hummel et al., 2013; Alexi et al., 2018, 2019), and have the advantage of providing an ecologically representative view of the human body. Real body images are usually acquired by photographing participants or sourced from the Internet. However, sourcing real body images using these methodologies can be challenging, particularly if full-length body images varying in body size and weight are required and can result in unwanted heterogeneity due to factors such as clothing, posture, and attractiveness, to name a few (Moussally et al., 2017).

CG body image stimuli provide an appealing alternative to real body stimuli, as they address many of these challenges. CG body stimuli are easily created through powerful yet relatively inexpensive CG imagery software that allows for the systematic manipulation of body characteristics, such as weight and size, thus creating precise and reproducible stimulus changes (Moussally et al., 2017). While CG stimuli can be highly human-like in appearance, distinct differences to real photographs are often noted (Farid and Bravo, 2012; Fan et al., 2014). For instance, within the field of face perception and computer graphics, research has identified unrealistic texture, illumination, and shading as factors that can decrease realism in CG stimuli (Fan et al., 2012, 2014; Crookes et al., 2015).

One concern, then, is that the reduction of visual realism in CG stimuli may consequently hamper processing mechanisms. In particular, a face perception study conducted by Crookes et al. (2015) examined how well CG face images tap face expertise abilities by comparing facial recognition abilities for own- and other-race faces across real and CG face images. Their findings revealed that recognition and discrimination accuracy of own-race faces were significantly diminished for CG faces, compared to real faces. The authors concluded that CG face stimuli may not entirely capture and tap face expertise (Crookes et al., 2015). This is likely to be because textural information is important in face recognition (Crookes et al., 2015). These findings are in concordance with other face perception studies, which have found that CG face images disrupt other facial processing mechanisms, such as perception of trustworthiness (Balas and Pacella, 2017) and face memory (Balas and Pacella, 2015).

Conversely, within the body perception literature, there have only been two published comparisons between CG and real bodies that we are aware of. The first by Tovée et al. (2012) and the second by Cornelissen et al. (2016). They found comparable results between CG and real body stimuli. However, in the first instance, attractiveness and health ratings were compared (Tovée et al., 2012), which are different dimensions of judgment from body size judgments. In the second instance, Cornelissen et al. (2016) compared the use of CG and real body images in the examination of body size sensitivity. However, this comparison used different Body Mass Index (BMI) size ranges between the CG (eight BMI ranges) and real (four BMI ranges) body stimuli (Cornelissen et al., 2016). For example, the largest BMI used in the real body condition was 26.5, versus 43 in the CG condition. Because the stimuli were not matched across each of the BMI groupings, a direct comparison of the two stimulus types appears problematic. Furthermore, if there are variations in body size judgments between CG and real stimuli, these are likely to be more pronounced in statistical extremes, where weight characteristics (e.g., visible emaciation, cellulite etc.) may be less well represented in CG stimuli. Additionally, the question regarding the effect of CG bodies on body size biases, such as serial dependence, is unexplored. Therefore, the current research sought to examine the efficacy of Poser-produced (Smith Micro Software, 2015) CG body stimuli to study body size judgments and whether using CG stimuli alters the magnitude and nature of two known biases that occur in the judgment of body size: regression to the mean and serial dependence.

Regression to the mean is a commonly reported bias in body size judgments (Cornelissen et al., 2016) and this bias occurs when judgments of stimuli are perceived to be closer to the mean of a set than they really are. In a study by Cornelissen et al. (2015), healthy controls with high psychological symptoms, including depression and eating and weight concerns, were shown to differ in their overall magnitude of body size estimations (i.e., they consistently overestimated their body size), but their slope of judgments (a common measure of regression to the mean) was unchanged (Cornelissen et al., 2015).

In contrast, serial dependence is a recently discovered bias in body perception, in which perceptual size judgments of stimuli are biased toward the size of previously viewed stimuli. It has been suggested that serial dependence might be particularly strong when the stimuli are relatively ambiguous with respect to the judgment to be made (Cicchini et al., 2018). It is proposed that this effect occurs because serial dependence works to increase the efficiency of our visual system and reduce overall noise (Cicchini et al., 2018). Serial dependence bias has been observed in the evaluation of a number of different stimuli, such as number (Fornaciai and Park, 2018), orientation (Fischer and Whitney, 2014; John-Saaltink et al., 2016), facial identity (Liberman et al., 2014), attractiveness (Xia et al., 2016), and gender (Taubert et al., 2016), and recently, in body size estimation (Alexi et al., 2018, 2019).

Importantly, serial dependence bias has recently been shown to be associated with eating disorder symptomatology (Alexi et al., 2019). However, this association was found using real body images. We do not know whether the same pattern of results would be evident with CG bodies. Previous research has highlighted the importance of social comparisons of oneself to other individuals’ bodies in predicting body image disturbances (Stormer and Thompson, 1996; Ridolfi et al., 2011). Given the reduced realism of CG body stimuli that is highlighted in the face perception literature, it is possible that the impoverished visual information might impact humans’ abilities to relate to CG stimuli in the same way that has been evidenced in social comparison research. If this were the case, the relationship between serial dependence and eating disorder symptoms would likely be underestimated. Alternatively, it may be that the distinction between CG and real body images is less imperative in body judgments (e.g., Cornelissen et al., 2015, 2016) than it is in other areas (e.g., Crookes et al., 2015), in which case we would expect to see a retention of the significant relationship previously observed between serial dependence and eating disorder symptoms (Alexi et al., 2019).

In order to examine the efficacy of CG body stimuli in the study of body size judgments and their biases, we utilized an established bodyline task (Alexi et al., 2018) for measuring body size estimation and the two aforementioned biases, but we modified the task by presenting CG body stimuli. While there have been some alternative findings in the literature regarding CG and real comparisons, the majority of research in CG imagery seems to indicate that there are subtle differences in the detection and judgment of CG compared to real images, with textural elements noted to play a role (Johnson et al., 2011; Tinwell et al., 2011; Balas and Pacella, 2015, 2017; Crookes et al., 2015). It seems reasonable to predict that textural information is also important in body perception, particularly so for extreme body sizes. For example, key body weight markers, such as visible bone structures in emaciation, or cellulite in obesity appear difficult to fully represent using synthetic textures. Therefore, we hypothesized that perception of CG body stimuli would be non-linear, with poor discrimination among extreme weight categories. Poorer discrimination is also predicted to result in larger body size estimation biases, namely regression to the mean and serial dependence (Alexi et al., 2018). Secondly, although speculative and assuming that our initial hypothesis is satisfied, we formed the intuitive hypothesis that CG body stimuli would reduce self-referencing abilities and result in a diminished relationship between serial dependence and eating disorder symptoms.

## Method

The current study was approved by the Human Research Ethics Committee of the University of Western Australia and completed in accordance with their rules, guidelines, and regulations. Participation was entirely voluntary and helped to form a component of participants’ undergraduate course credit. All participants provided written informed consent prior to completing the experiment and were debriefed in full following completion of the experiment. Additionally, this study was completed in parallel to the Alexi et al. (2018) study, which established serial dependencies in body size estimations using the bodyline task with real bodies. Therefore, the same sample of participants from Alexi et al. (2018) completed the tasks in the current study.

### Participants

One hundred and six female undergraduate psychology students from The University of Western Australia participated in the study. Two participants’ data were removed due to failure to follow the task instructions. A third participant’s data were removed due to a computer malfunction. Therefore, the analyses outlined below were completed using the remaining participants’ data (*N* = 103). The age of the participants ranged from 17 to 25 years (*M* = 18.88, SD = 1.65) and participants’ BMI ranged from 16.23 to 43.99 (*M* = 22.22, SD = 3.93). We restricted our sample to females aged between 17 and 25 due to the high prevalence of eating disorders in this sample (The Butterfly Foundation, 2012).

### Materials

#### Stimuli

Each of the CG body images was created on computer-generated (CG) software, Poser version 11 (Smith Micro Software, 2015). Poser software was utilized in the current study for consistency with previous research that regularly employed this software to develop body stimuli (e.g., Glauert, 2008; Nikkelen et al., 2012; Cho and Lee, 2013). Poser software contains multiple body weight dials, such as the “thin” or “heavy” dials, which can be reduced or increased incrementally when creating body stimuli. Adjusting these dials alters the body weight and size of a chosen CG figure consistent with the descriptor of the dial. The dials “thin,” “emaciated,” “heavy,” and “rubenesque” were chosen for creating our CG body stimuli as they were the most relevant dials to use and produced body types and sizes most consistent with previously established real body images (Alexi et al., 2018).

##### Pilot Study to Calibrate Size Range of Computer-Generated Stimuli to Prior Real Body Images

In our earlier work involving real body images, we presented size body size categories (Alexi et al., 2018, 2019). We decided to design CG stimuli that covered the same overall size range as in our previous work. Accordingly, we used Poser software (Smith Micro Software, 2015) to create two sets of CG body images; the first set of three images represented thin endpoint (category 1) real body images and the second set of three matched the heavy (category 7) real body images used by Alexi et al. (2018, 2019). Each set of three body images varied slightly in weight, yet still remained representative of their respective category. We manipulated the “thin” and “emaciated” dials to create the category 1 representative body images. In contrast, the “heavy” and “rubenesque” dials were adjusted to form the category 7 representative body images. Each of the CG body images was created using the CG model “Alyson.” The body images were clothed in underwear in order to match previous body images (Alexi et al., 2018, 2019) and permit view of key body weight markers (e.g., hollowing skin, cellulite etc.).

Using the abovementioned CG body images, a pilot study with five participants was conducted to establish the best matching CG endpoint body categories 1 and 7, with respect to the real body endpoint categories from Alexi et al.’s (2018) study. Participants were first shown the three exemplar category 1 CG body stimuli and each of the five real category 1 bodies used by Alexi et al. (2018). Participants were then asked to choose which of the three CG body stimuli best matched the real category 1 bodies. Using this same methodology, participants then selected the closest CG match to the real category 7 body images.

##### Creation of the Computer-Generated Body Continuum

Using the data from our pilot study, the best matched category 1 and 7 CG body images (and body weight dial values) were determined. This selection was based on an inter-rater agreement of ≥60%. Following selection of the best matched category 1 and 7 CG bodies, the rest of the full body continuum was created in Poser (Smith Micro Software, 2015). This was done by varying the dial values, as determined by the pilot study, in equal linear steps along the continuum. As the dials “thin” and “heavy” were considered exact opposites of the body weight continuum, we used the difference value between the “thin” and “heavy” dials to divide the two dials into equal increments along the body continuum. The “emaciated” and “rubenesque” dials were also considered to reside on opposite ends of the body weight continuum. Therefore, we applied the above method to the “emaciated” and “rubenesque” dials. Hence, the final CG body continuum resulted in the use of the “thin” and “emaciated” dials for the “thinner” end of the CG body continuum (categories 1 to 3) and then transitioned into the use of the “heavy” and “rubenesque” dials for the “heavier” end of the CG body continuum (categories 4 to 7). The two sets of dials (“thin” and “heavy” and “emaciated” and “rubenesque”) increased in equal linear steps along their respective ends of the continuum, which resulted in a continuous transition from thin to heavy.

Once each of the seven body category dial values had been determined using the above methodology, we created the rest of the CG body image database. This resulted in the creation of 35 CG body images (five images per category) for use in the bodyline task. These body images ranged from extremely underweight to extremely overweight, along the body continuum. The total number of CG body images and body categories were chosen to match the prior real body image database (Alexi et al., 2018, 2019).

**Figure 1:**
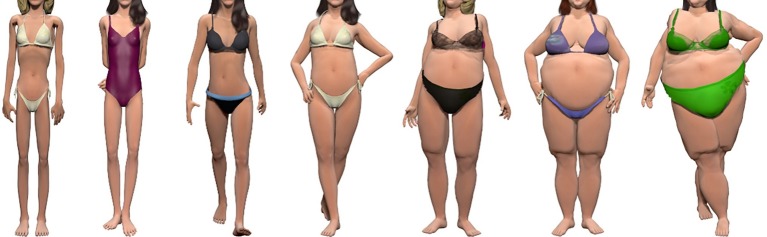
Example CG body image categories from one (left) to seven (right), which were used in the CG bodyline task. The depicted body images were created with CG imagery software, Poser Version 11 (Smith Micro Software, 2015).

We ensured that our set of CG body images matched the previously established and validated real body images (see Alexi et al., 2018) on identity and clothing, where possible. We did this by giving the final CG body images’ variations in pose and clothing to closely match prior research (Alexi et al., 2018, 2019). While we varied the skin tones of the CG bodies, we used the same model type (model “Alyson”) for each of the CG body stimuli. This allowed each of the CG body images to represent a unique identity, and match, as close as possible, the real body images first used in Alexi et al. (2018). As in that study, the images were cropped on Adobe Photoshop to display the whole body but omit the face. The omission of face stimuli in this experiment was deliberately implemented to ensure that the attractiveness of the stimuli did not bias participants’ judgments of size. See Figure 1 for an example of the final seven CG body image categories.

Six CG body anchors (three of which represented an extremely underweight body anchor and three of which represented an extremely overweight body anchor) were also generated in Poser (Smith Micro Software, 2015) using the “thin” and “emaciated” and “heavy” and “rubenesque” dials, respectively. These body anchors were generated to match the previously established real body anchors on size (Alexi et al., 2018) and to be used as off-scale body anchors denoting the two ends of the visual analogue scale in the bodyline task. They were created to be more extreme in weight than any of the CG body categories, analogous to the real body image database (Alexi et al., 2018). Using the same methodology as in our pilot study, the same five participants were asked to select which one of the three extremely underweight and extremely overweight CG body anchors best matched the real body anchors. The final CG body anchors were selected based on inter-rater agreement of >60%. The CG body anchors were also matched to the real body anchors on identity, clothing, pose, and stance.

#### Eating Disorder Examination – Questionnaire 6.0

The Eating Disorder Examination – Questionnaire 6.0 (EDE-Q) is a self-report eating and weight behaviors questionnaire, which was based on the original interview format questionnaire (Fairburn and Beglin, 1994). The EDE-Q comprises 28 items in total, 22 items of which explore overall attitudinal components of eating disorder symptomatology (Mond et al., 2004). The 22 items form the subscales of Restraint (5 items), Eating Concern (5 items), Weight Concern (5 items), and Shape Concern (8 items). Restraint and Eating Concern subscales measure abnormal eating behaviors, while Weight and Shape Concern subscales examine negative body image, across the preceding 28-day period (Hilbert et al., 2012). Respondents answer across a 7-point, forced choice, Likert rating scale (0 = *complete absence of feature* to 6 = *acute presentation of feature*) (Hilbert et al., 2012). The remaining six items measure information relevant for diagnosing an eating disorder, such as self-induced vomiting. Reliability and validity of the EDE-Q are well established (Mond et al., 2004, 2006). The Cronbach’s alphas for the EDE-Q in our sample were: 0.80 (Dietary Restraint), 0.77 (Eating Concern), 0.91 (Shape Concern), and 0.84 (Weight Concern).

### Procedure

Participants were seated in a quiet room facing a computer screen, keyboard, and mouse, which the experiment was completed with. The experiment was conducted on an Asus branded PC running Matlab (The MathWorks Inc., 2013) and the Psychophysics Toolbox (Brainard, 1997). The CG body stimuli were shown on a Viewpixx branded PC monitor with a resolution, size, and luminance consistent with the previous “bodyline” study by Alexi et al. (2018). The size and contrast of the stimuli were also consistent with Alexi et al. (2018).

Participants were given instructions regarding the experiment and were then asked to read an information sheet and sign the corresponding consent form. All participants gave written informed consent and were instructed in detail prior to completing the experimental tasks. Following this, participants completed an established bodyline task, which has been shown to measure both, regression to the mean and serial dependence biases (Alexi et al., 2018). The bodyline task in this experiment was adapted to consist of the CG body images created for this experiment.

During the bodyline task, participants were required to judge the weight of various body stimuli, ranging from underweight through average-weight to overweight, using a continuous visual analogue scale (VAS). Participants recorded their responses by left-clicking the mouse along the VAS at the bottom of the screen. The VAS was an unmarked line scored linearly from 1.0 to 7.0, and was present throughout the bodyline task. A CG anchor body image was presented beyond each end of the bodyline scale, demarcating the two extreme weights: extremely underweight and extremely overweight. These anchors were more extreme than any of the body images shown throughout the experiment.

**Figure 2:**
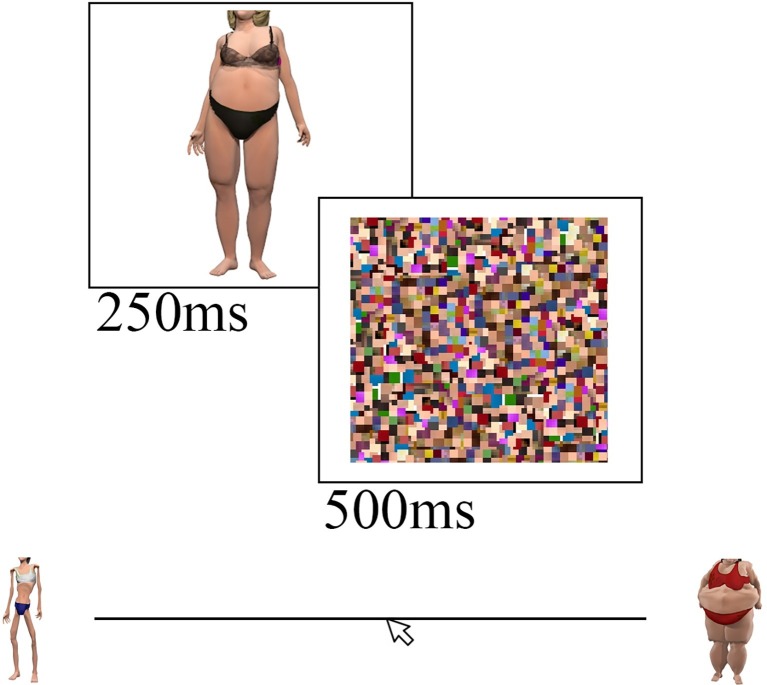
A visual representation of the CG bodyline task. The bodyline task required participants to judge the size of CG body stimuli that were presented for 250 ms, followed by a visual noise mask for 500 ms. Participants recorded their body size estimations by left-clicking their mouse along the bodyline, which showed an extreme body anchor displaced from each end of the scale. The anchor images were more extreme in size than all of the body images presented throughout the bodyline task. The bodyline was continuously presented throughout the task. The body images presented here were shown in the experiment and were created using CG imagery software, Poser (Smith Micro Software, 2015).

Participants first completed 14 practice trials, followed by three blocks of 50 trials. Body stimuli were presented in a fixed order across all subjects, identical to the order presented by Alexi et al. (2018, 2019). Each of the body images were presented for 250 ms, followed by a random-noise mask, comprised of scrambled fragments of the CG body images, for 500 ms. The noise mask was implemented to interrupt visual processing of the stimuli and to prompt participants for a response. See Figure 2 for a visual depiction of the bodyline task. Upon completion of the bodyline task, participants completed the EDE-Q. Participants’ own height and weight were then measured in order to obtain an estimate of participants’ Body Mass Index (BMI).

## Results

The results section first includes a description of the data cleaning process. Secondly, we outline the bodyline, regression to the mean and serial dependence data to provide an examination of our main hypothesis. We then go on to examine our second hypothesis by reporting the correlational analysis between our two perceptual biases and EDE-Q. The data from our pilot study and main results were analyzed using SPSS statistical software and Graphpad Prism software.

### Data Cleaning and Outlier Removal Process

Before data analysis, the EDE-Q and BMI variables were screened for normality using the criteria of skew < |2.00| and kurtosis < |7.00| (Curran et al., 1996). Using these guidelines, our EDE-Q variable was associated with an appropriate level of normality for the purposes of our analyses. However, our BMI variable was associated with a high level of skew (2.19) and kurtosis (8.87), which appeared to be driven by an outlier. Therefore, we analyzed the BMI variable using the outlier criterion method of three standard deviations above and below the mean (Howell, 1998). Using this criterion, one outlier was identified and subsequently winsorized (Reifman and Keyton, 2010; Ghosh and Vogt, 2012). Following revision of the outlier through winsorizing processes, skew and kurtosis of the BMI variable were reduced to 1.16 and 1.87, respectively. See Table 1 for revised BMI descriptive statistics.

**Table 1:** Descriptive statistics associated with participant BMI and EDE-Q subscale and global scores.

	BMI	EDE-Q R	EDE-Q EC	EDE-Q SC	EDE-Q WC	EDE-Q G
*M*	22.12	1.43	1.00	2.54	2.16	1.78
SD	3.48	1.26	1.02	1.49	1.51	1.17
Min.	16.23	0	0	0	0	0
Max.	33.73	5.40	5.40	6.00	5.60	5.40

*M, mean; SD, standard deviation; EDE-Q R, Eating Disorder Examination Questionnaire Restraint Subscale; EDE-Q EC, Eating Disorder Examination Questionnaire Eating Concern Subscale; EDE-Q SC, Eating Disorder Examination Questionnaire Shape Concern Subscale; EDE-Q WC, Eating Disorder Examination Questionnaire Weight Concern Subscale; EDE-Q G, Eating Disorder Examination Questionnaire Global Score*.

### Computer-Generated Body Size Estimation and Regression to the Mean

**Figure 3:**
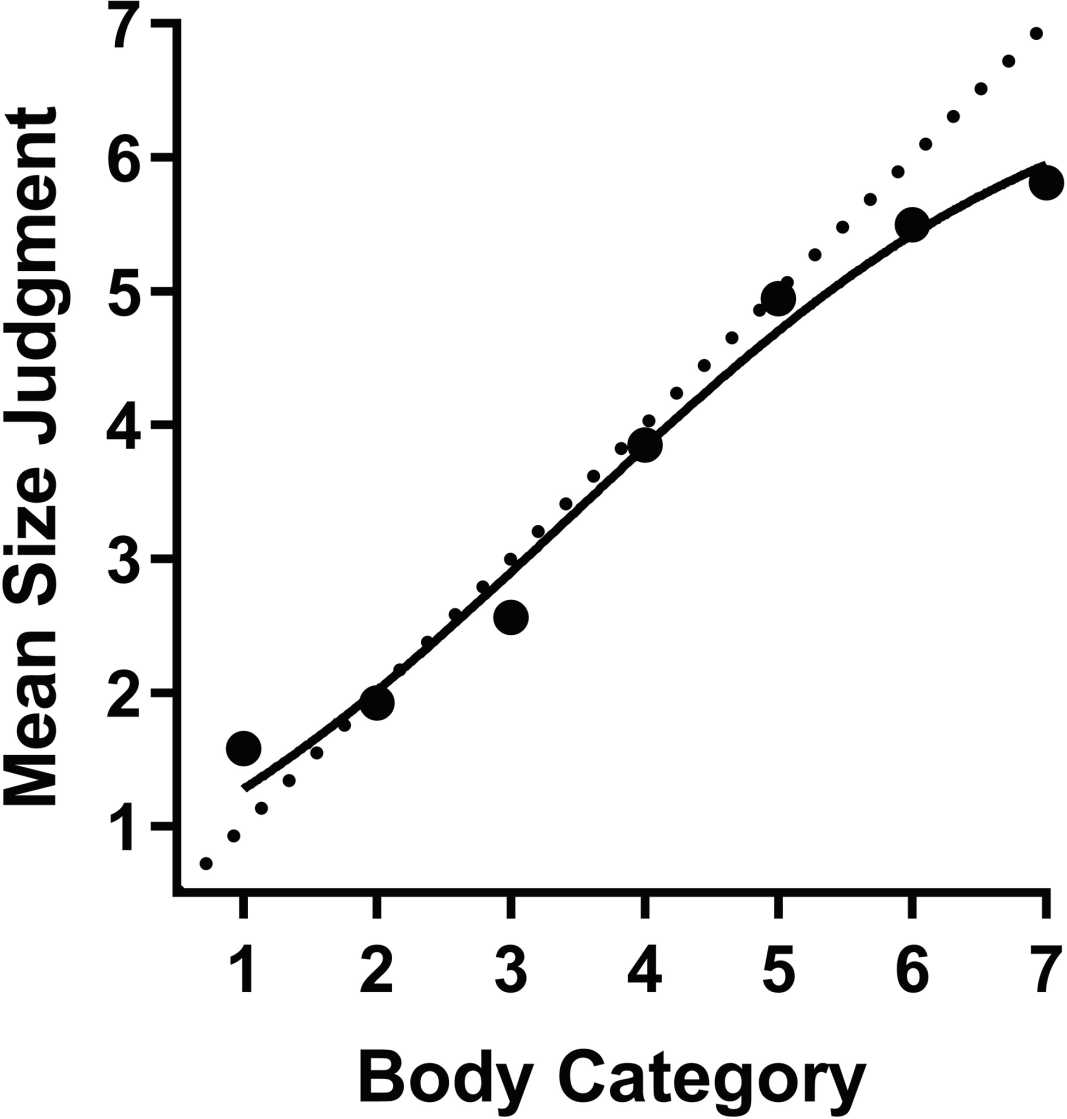
Visual depiction of the bodyline task data for CG body images. Data show average body size judgments across the seven body image categories. The dotted diagonal line shows unbiased, veridical judgment of the body categories. Error bars depicting SEM are plotted.

In order to address our main hypothesis regarding the judgment of CG body stimuli, we first report the mean body size judgments of each category when participants were presented with CG body stimuli (see Figure 3). These data relate to our first perceptual bias, regression to the mean. In the next section, we will report on the other perceptual bias, serial dependence.

The pattern of responses in the CG bodyline data appears to be a non-linear sigmoidal shape (see Figure 3). Visually, this is reflected by smaller perceived size changes between categories 1–3 and 5–7, while there is a disproportionately large perceived size change between categories 3 and 5. This non-linear interpretation is supported numerically and statistically. Numerically, we note that 56% of the scale is being used to represent the size change across near to average size bodies (categories 3–5) while only 44% of the scale is used to represent the size change across the remaining body categories, despite being double in number and bearing in mind that we specifically constructed uniform, linear increments across categories, in Poser. Statistically, the non-linear trend of the data was confirmed by a comparison of fits analysis. This analysis compared a linear fit, as per Alexi et al. (2018), and a non-linear (Cumulative Gaussian) fit, to the CG bodyline data. The analysis revealed statistical support for a non-linear fit, *F*(1, 718) = 112.6, *p* < 0.0001. Finally, we note that the non-linear fit was an excellent fit to the data (*R*^2^ = 0.91). We conclude then that the estimation of body size for CG bodies created by our Poser methods is non-linear.

Next we consider the strength of the regression to the mean bias for CG bodies. Fitting a linear regression slope to the bodyline data can provide an estimate of a regression to the mean bias if the slope observed is less than 1.0 (assumed as veridical perception) (Alexi et al., 2018). Applying a linear fit to the CG body data also produced data consistent with regression to the mean (Slope = 0.79, 95% CI: 0.63–0.96). However, since a linear fit is not appropriate for the CG data, this estimate may be problematic to interpret in relation to other regression to the mean estimates.

### Computer-Generated Body Size Estimation and Serial Dependence

**Figure 4:**
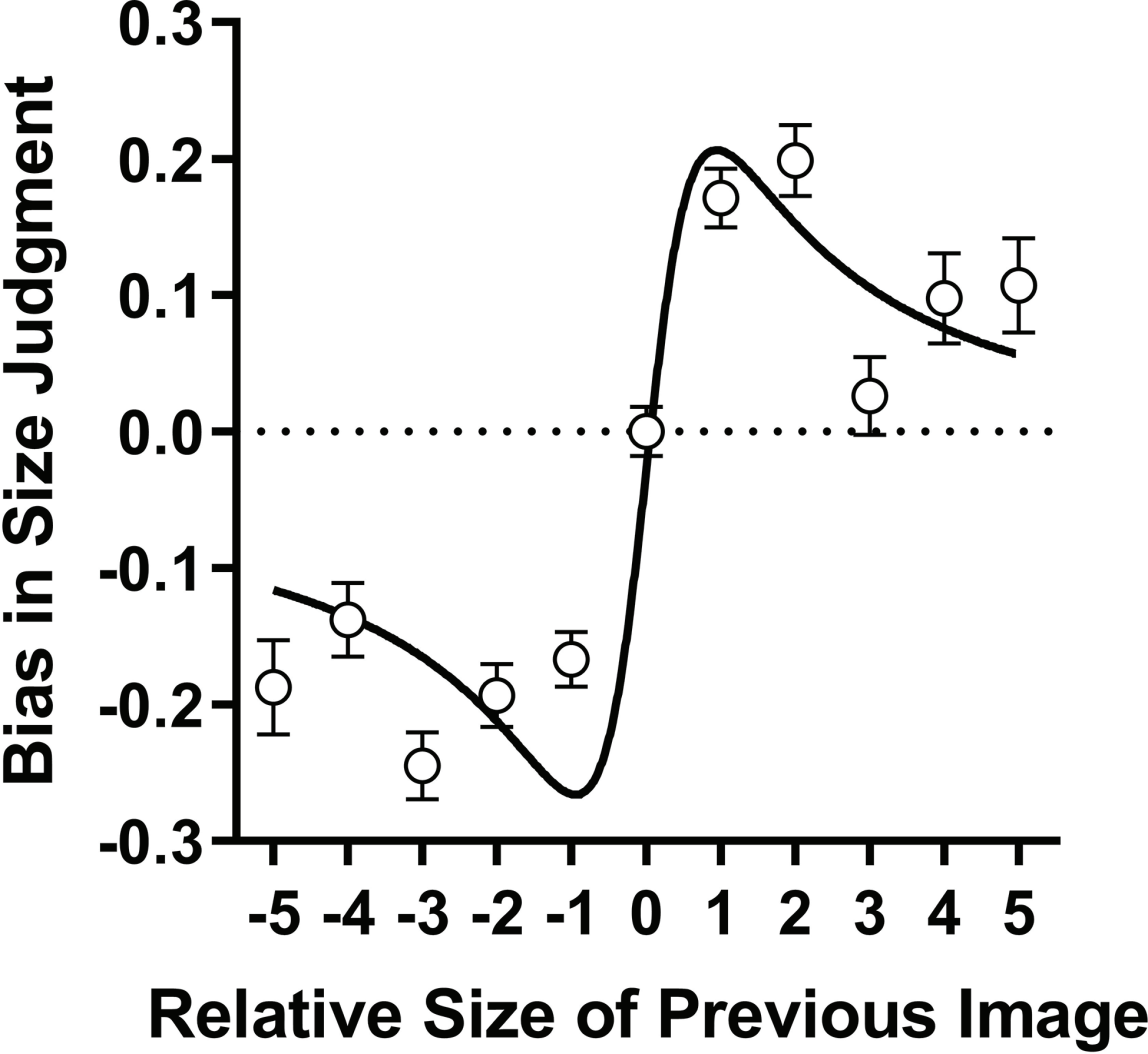
Serial dependence bias in body size estimation using CG body images. The data display the average bias in the perceived size (difference between perceived and physical size of the body images), as a function of the size of the previously viewed body image. Error bars depict ± 1 SEM. The solid black curve demonstrates the prediction of the unconstrained Kalman-filter model described in Alexi et al. (2018). The dotted horizontal line shows zero bias in size judgments.

As the second part of our main hypothesis, we investigated the presence of serial dependence for CG body stimuli. Serial dependence was calculated in accordance with the procedures outlined by Alexi et al. (2018). Figure 4 plots the average bias in body size judgments (i.e., the difference between responses and physical stimuli) along the vertical axis, as a function of the size difference of the previously viewed body image, along the horizontal axis. As would be expected, no bias was found for trials where the previously seen body was the same category as the current body (location zero on both, horizontal and vertical axes). Note, data residing on the dotted horizontal line indicated unbiased or veridical body size perception. As can be seen, the CG body data were consistent with serial dependence. That is, participants’ size estimates were biased toward the previously seen body. Specifically, participants were biased to see bodies as thinner than they actually were when they were preceded by thinner body images (see lower left quadrant of Figure 4) and the reverse was true for the larger body categories (see upper right quadrant of Figure 4). The CG body data were well fit (*R*^2^ = 0.86) by a Kalmann-Filter model, as used by Alexi et al. (2018).

### Correlational Findings Between Body Size Biases and Eating Disorder Examination – Questionnaire 6.0

Lastly, we examined our final hypothesis, relating to whether the biases observed in the CG body data were associated with EDE-Q. See Table 1 for the descriptive statistics associated with participant EDE-Q subscale and global scores.

We report the correlational findings in the order that we presented the body size biases above. A partial correlation was first conducted between participants’ regression index (i.e., participants’ magnitude of regression to the mean bias) and their associated global EDE-Q score, controlling for BMI. Participant regression indexes were determined using the same methodology as first described by Alexi et al. (2018). The correlation in this study revealed a non-significant association, *r*(100) = −0.16, *p* = 0.120, suggesting a non-significant relationship between regression to the mean bias and eating disorder symptomatology.

Next, we conducted a partial correlation analysis between participants’ magnitude of serial dependence and global EDE-Q, while controlling for BMI. The results from this partial correlation yielded a non-significant association, *r*(100) = −0.01, *p* = 0.903. In a previous study by Alexi et al. (2019) who used real body stimuli, a significant association between serial dependence magnitude and global EDE-Q was found [*r*(59) = 0.28, *p* < 0.05]. The discrepancy in findings between Alexi et al.’s (2019) findings and our current research may well be explained by the use of the CG body stimuli. The interpretation of this result is framed within the context of reduced self-comparisons for CG bodies in the “Discussion” section below.

## Discussion

The current study investigated body size estimations of CG body stimuli. We firstly hypothesized that the perception of sizes of CG bodies would be non-linear, leading to increased perceptual biases, namely regression to the mean and serial dependence. Secondly, we did not expect to find a significant correlation between serial dependence magnitude and eating disorder symptoms when judging CG body images, due to the hypothesized importance of self-reference when judging bodies.

Our findings provided support for our first hypothesis, by showing non-linear body size estimation when participants judged CG body images. The non-linear pattern of the CG bodyline data makes it problematic to provide a sensible estimate of regression to the mean using the traditional linear slope analysis. We can say, however, that as predicted, participants clearly found it difficult to discriminate between the more extreme CG size categories (i.e., categories 1–3 and 5–7), compared to the same real body categories in our previously published work (Alexi et al., 2018), which showed linear discrimination. This finding accords with previous research showing that discrimination and recognition of faces is also significantly reduced for CG faces (Crookes et al., 2015). Therefore, we take the results of our CG bodyline task to suggest that the use of CG body stimuli reduces discriminability between body categories. We hypothesize that this is due to the impoverished representation of textural elements in CG body stimuli. For instance, the smoothing functions in CG Poser software do not well represent the subtle textural differences in skin, such as cellulite, hollowing skin surfaces, or visible bones, which observers may use as markers of body weight.

We should point out that two other studies (i.e., Tovée et al., 2012; Cornelissen et al., 2016) that compared the use of real and CG bodies reached alternative conclusions to the current study, as described in the “Introduction” section. This discrepancy in findings may be a result of the type of judgment asked of participants in Tovée et al.’s (2012) study or may reflect a lack of sensitivity to the differences in CG and real bodies by using differing BMI ranges to compare performance, as was done in Cornelissen et al.’s (2016) study. Alternatively, as previously suggested, it may be that the specific software we used here resulted in stimuli that do not represent body size changes as precisely as other versions or software used in these other studies.

Analysis of the second perceptual bias, serial dependence, provided strong support for our main hypothesis. We found that the serial dependence bias for CG body images was up to 50% larger than the size of the serial dependence effect demonstrated by Alexi et al. (2018) for real body images. This finding can likely be explained by the conditions that are known to increase serial dependence biases. Specifically, serial dependence works to stabilize perception of ambiguous stimuli/scenes, resulting in largest serial dependencies when there is high stimulus uncertainty (Cicchini et al., 2018). Considering that our data suggest poorer (non-linear) discrimination for CG bodies compared to real bodies, we would expect this to manifest in larger serial dependencies for CG than real bodies (Cicchini et al., 2018). Our finding of a larger serial dependence bias for CG bodies accords with this view and is consistent with previous findings (Cicchini et al., 2018). This result signals the importance of using CG body stimuli with caution, as they are more poorly discriminated between than real bodies and participants show larger errors, in the form of larger serial dependence biases, when judging their size.

We next examined the association between the two measured perceptual biases and eating disorder symptomatology. We found no evidence for an association between regression to the mean and eating disorder symptoms. This finding corroborates previous findings by Cornelissen et al. (2015), who found that psychological symptoms had an effect on the overall magnitude of judgments but not the gradient of judgments (i.e., regression to the mean). However, critically, our results showed that eating disorder symptoms were also not significantly related to serial dependence bias for CG bodies. This finding is of particular interest, considering recent evidence for a significant association between eating disorder symptomatology and serial dependence bias for real body images (Alexi et al., 2019). One possible explanation for the absence of such a relationship in the perception of CG bodies is that this stimulus class may have reduced participant self-comparison to their own bodies. Social comparison theory suggests that individuals compare themselves to others in order to make judgments about their own characteristics (Bessenoff, 2006; Franzoi et al., 2011). In fact, a study conducted by Stormer and Thompson (1996) found that social comparison to others’ bodies was a fundamental contributor to bodily dissatisfaction. Therefore, it is possible that the use of CG bodies led to a reduction in the comparisons of oneself to the CG body images, which may have consequently diminished the effect of individual differences in serial dependence. Together, the correlational findings observed in serial dependence provides support for our second hypothesis as it shows a diminished relationship between EDE-Q and body size biases for CG bodies.

We offer two likely explanations for our collective findings of poorer discrimination and increased errors in the body size estimation of CG bodies. Firstly, as we and others (Crookes et al., 2015) have proposed, poorer discrimination for CG stimuli may reflect an objective lack of relevant feature information in this stimulus. Research has shown that despite the element of realism that CG imagery can generate, observers are still able to identify a CG image from a real image, suggesting that CG images may be unable to fully capture the nuances of real-life stimuli (Johnson et al., 2011; Farid and Bravo, 2012). This failure to fully replicate real bodies leads to less realistic body categories. This may be particularly true for very underweight and very overweight bodies, where skin texture and detailed feature characteristics are particularly important. However, there is a fine line between CG realism and complete human-likeness (Tinwell et al., 2011). Generating CG stimuli that are hyper-realistic may result in the “uncanny valley” effect – a phenomenon whereby observers describe aversive reactions toward hyper-realistic CG characters, due to subtle oddities in their appearance (Tinwell et al., 2011). Importantly, the “uncanny valley” phenomenon demonstrates how attune observers are to the subtle variations in CG imagery, therefore highlighting the importance of providing information-rich and accurate CG body images.

Secondly, our findings may instead reflect a lack of participant exposure to, and familiarity with, CG bodies (Crookes et al., 2015). It is well known that humans develop an expertise in judging human faces (Crookes et al., 2015). Neuropsychological and imaging findings suggest that it is likely that humans develop a similar expertise for perceiving and judging bodies, which is strengthened and fine-tuned over time and with continued exposure (Downing et al., 2001; Ishizu et al., 2010; Downing and Peelen, 2016; Gillmeister et al., 2019). Given humans view real, not CG bodies, on a daily basis, it is conceivable that our results may instead reflect a reduced expertise for CG bodies. Therefore, it may be particularly important to discern whether our results reflect reduced familiarity with CG bodies or are the result of a poorer “make up” of the CG bodies used in our task (e.g., less textural information). Future studies may explore the effect that additional training and exposure to CG bodies has on the accuracy of body size judgments (Balas and Pacella, 2015). Alternatively, it may be worth comparing the accuracy of body size estimates between participants who already have substantial exposure to CG imagery (e.g., individuals who frequently play video games involving CG bodies) to those who do not.

Our results highlight the shortcomings of using CG body stimuli in body size estimation tasks. However, we acknowledge the boundaries of our findings are limited to stimuli created with Poser version 11 software. Additionally, given the fast-paced nature of technological advancements in computer graphics, it is important to keep in mind that these results may differ with the use of other Poser versions or with other CG software and in the future with more advanced technology. In fact, some of these advancements are beginning to take shape already. In particular, recent research has begun to incorporate the use of hybrid based body stimuli (Stephen et al., 2018). This stimulus type typically involves obtaining photographs of real bodies under controlled conditions (e.g., standardized lighting, clothing, and photograph angles), which are then systematically manipulated across body weight biomarkers, to create body images that vary along the body weight continuum. This type of manipulation is proposed to simulate typical body weight changes and therefore achieve more realistic transformations in fat mass than previous methods, which simply widened or “stretched” images to achieve the appearance of body weight changes (Stephen et al., 2018). Hybrid body stimuli are advantageous in that they incorporate real body images, which are ecologically sound, while also achieving standardized body weight increments, using morphing software. Additionally, new stimulus methodologies have started to incorporate the use of 3D body scanning equipment to create a mesh of participant’s own bodies, which are then used to generate personalized CG body avatars (Cornelissen et al., 2017). Using this type of stimulus class may enhance ecological validity by stimulating the act of looking in a mirror when judging body size (Cornelissen et al., 2017). Three-dimensional CG body stimuli have also recently been created and used within the virtual reality context to examine body image distortions (Ferrer-Garcia et al., 2017; Serino et al., 2019). While these body images are computer-generated, they provide an increased element of realism in their three-dimensional presentation, which may better highlight certain body weight markers (e.g., stomach and thighs). Future research would benefit from understanding whether these stimuli types are more ecologically valid and efficacious than traditional CG stimuli.

In conclusion, the current study examined the effect of CG body images on body size estimation and their associated estimation biases: regression to the mean and serial dependence. Our results suggested poorer discriminability among the CG bodies and larger body size judgment errors, which were demonstrated by larger serial dependencies. Taken together, our results highlight the importance of using caution when employing CG body stimuli in the study of body size estimation and its biases. Furthermore, our findings suggest that care should be taken when interpreting the findings of studies that do use CG bodies. Our combined findings provide useful information to researchers seeking to develop experimental tasks that require the use of body images.

## Data Availability Statement

The datasets created and analyzed during the current research are available from the corresponding author on request.

## Ethics Statement

The studies involving human participants were reviewed and approved by Human Research Ethics Committee of the University of Western Australia. The patients/participants provided their written informed consent to participate in this study. Written informed consent from the participants’ legal guardian/next of kin was not required to participate in this study in accordance with the national legislation and the institutional requirements.

## Author Contributions

JA, JB, RP, and NK designed the study. Testing and data collection were performed by JA, KD, and DC. JA and JB analyzed the data and drafted the manuscript. All authors provided critical revisions and approved the final manuscript for submission.

### Conflict of Interest

The authors declare that the research was conducted in the absence of any commercial or financial relationships that could be construed as a potential conflict of interest.
